# Effects of Electrical Stimulation on Meat Quality of Lamb and Goat Meat

**DOI:** 10.1100/2012/574202

**Published:** 2012-04-19

**Authors:** Omer Cetin, Enver Baris Bingol, Hilal Colak, Hamparsun Hampikyan

**Affiliations:** ^1^Department of Food Hygiene and Technology, Faculty of Veterinary Medicine, Istanbul University, Avcilar, 34320 Istanbul, Turkey; ^2^The School of Vocational Studies, Beykent University, Buyukcekmece, 34500 Istanbul, Turkey

## Abstract

Effect of various voltage of electrical stimulation (ES) on meat quality of lamb and goat was investigated by using a total of 36 animals at 3–5 years old. Constant 50 Hz frequency and 50, 100, and 250 V, 90 sec of ES were administered to 1/2 carcasses and were examined according their textural, physicochemical, and sensorial characteristics. ES decreased the pH values of lamb and goat meat, and accelerated the rigor mortis (*P* < 0.05). Additionally, ES enhanced the water activity, water-holding capacity, and drip loss of both animals. Shear force varied between lamb and goat meat, and tenderness was improved depending on voltage range used (*P* < 0.001). ES caused difference in instrumental colour (CIE *L* ^∗^, *a* ^∗^, *b* ^∗^) values of lamb and goat meat compared with the control groups (*P* < 0.05) during aging period at 4°C. Sensorial characteristics were also improved with various levels of ES treatments. In conclusion, ES had positive effects on meat quality of lamb and goat, in contrast to undesirable consumer preferences.

## 1. Introduction

Organoleptical properties such as colour, texture, and flavour are important criterions determining the meat quality. These properties are related to age, gender, race, nutrition, nursing, anatomical, and technological characteristics [1, 2].

Electrical stimulation (ES) is an innovation being used in the meat industry to increase meat tenderness and colour of beef, lamb, and goat carcasses [3–5]. ES is a procedure that depends on electric current passing through hot carcass immediately after slaughtering [6]. Passing of voltage lower than 100 volts is known as low-voltage electrical stimulation (LVES) and treatments with higher than 100 volts as high-voltage electrical stimulation (HVES). Although increase in efficacy is reported with increase in voltage, it is risky for workers to stimulate with high voltage. Nowadays, high-voltage electrical stimulation studies are limited [2, 7]. The electric current flowing through the muscle tissue causes pH decline by increasing postmortem glycolysis. It also partially decreases the microbial total count of the carcasses by preventing cold shortening and improving some quality parameters such as colour, tenderness, and flavour [2, 3, 8].

Sheep and goat meat are important sources of protein in our country as in the world [9–11]. Approximately 159,000 tons of the total red meat production of slaughtered animals (780,718 tons) in 2010 was obtained from small ruminants. According to this, about 20% of the total production in 2010 was produced from sheep and goat meat [12].

Goat meat is tough as it contains less fat, so it is not generally preferred by consumers. Sheep meat is more tender than goat's meat; however, with the age of the sheep, a typical mutton flavour decreases its preferences by consumers [9, 11]. There are various applications to avoid the limiting properties of goat's and sheep's meat; in our days, low- and high-voltage ES applications become an ideal application area to minimize these negative effects of both meats [3, 5, 13].

This study was conducted to investigate the effects of various voltage of electrical stimulation on meat quality of lamb and goat meat. 

## 2. Materials and Methods

### 2.1. Materials

A total of 18 Kivircik breed lambs and 18 Malta goats between 3 and 5 years old, which are at the same age period, same breed, and are subjected to the same feeding process, were processed by the approval of the Ethic Committee of the Istanbul University, Turkey (approval number: 58/26.05.2011).

### 2.2. Preslaughter and Slaughtering Process

Animals were transported to the slaughterhouse from farm one day before the slaughter and were held in different shelters. During this period, they were provided with *ad libitum* water and kept without feed for 24 h before slaughter. After the rest period, animals were put through health detection and sent for slaughter without any stress.

The animals were stunned applying 220–250 V, 1.0–1.3 A, and 1–3 seconds of electroshock and then slaughtered by Halal method. Following exsanguinations, dressing, and evisceration processes, the carcasses were halved by splitting along the vertebral column approximately in 30–45 min postslaughter period, and one side of the carcasses was kept for ES treatment.

Both animal groups (lamb and goat) were divided into three subgroups according to ES. Half-carcasses were stimulated with 17 impulses (1.8 s duration each, with a 1.8 s interval between pulses) constant voltage (AC) at 2.5 amps, and 50 Hz. Half-carcasses in the first group were stimulated with low voltage (LVES; 50 V, 50 Hz for 90 s), the second ones were stimulated with medium voltage (MVES; 100 V, 50 Hz for 90 s), and the third ones were stimulated with high voltage (HVES; 250 V, 50 Hz for 90 s). ES was applied to the right sides of each carcass, and the corresponding left carcasses were used as controls (no electrical stimulation, NES). After electrical stimulation, all the half-carcasses were maintained at 0–4°C air flow 1–1.5 ms^−2^.

### 2.3. Sampling and Measurements

During the first 24 hours, carcasses were held at the cold chain (4°C) and at the end of the first day; samples were taken from the back (*M. longissimus dorsi*, LD) muscles of splitted carcasses and were examined according their textural (Warner-Bratzler shear force), physicochemical (instrumental colour, pH, water holding capacity (WHC), water activity (*a*_*w*_), and drip loss (DL)), and sensorial characteristics (colour, odour, appearance, and tenderness).

### 2.4. Determination of pH

At 1, 3, 6, and 24 h of postmortem, pH of meat samples was measured using a portable pH meter (WTW pH 340i with a probe SenTix, Weilheim, Germany). The mean of three measures in each sample was evaluated as pH value [14].

### 2.5. Determination of Water Activity (*a*_*w*_)

Water activity measurement was carried out using a water activity device (Hygrometer-Lufft, Fellbach, Germany). A 20 g of meat sample was placed in the cup of the instrument, and at the end of 3 h, values were recorded. The meter was calibrated using the manufacturer's standards. All water activity measurements were performed at room temperature (25 ± 2°C) [14]. 

### 2.6. Determination of Drip Loss (DL)

Following the slaughter, the carcasses were weighed as a whole on the 0, 1, 3, and 7 days, while they were kept in the fridge at 4°C. Thereby, the drip loss was evaluated by subtracting the values from the previous day's values using a digital scale [15].

### 2.7. Determination of Water-Holding Capacity (WHC)

To measure the water-holding capacity, 300 mg meat samples were collected from control and treated sides of the carcasses on the 1, 3, and 7 days of the postmortem phase and placed on Whatman no. 1 filter paper. The samples were kept between glass slips and under a fixed weight of 1 kg for 20 minutes. At the end of the waiting period, the filter paper was taken. The impressions released by the water were measured using millimetric paper and calculated by appropriate formulas [16],

(1) $$\begin{array}{cc} \text{Water}\;\text{holding}\;\text{capacity} & =\frac{\;\text{Range}\;\text{of}\;\text{dispersion}\;\left(\text{c}{\text{m}}^{2}\right)}{\text{total}\;\text{area}\;\left(\text{c}{\text{m}}^{2}\right)}. \end{array}$$

### 2.8. Instrumental Colour Measurement

The surface colour of meat samples at five different locations on each muscle and averages were determined at each sampling day in terms of L* (lightness), a* (redness), and b* (yellowness) values using colour difference meter [17]. Samples were placed in a special cup, which fitted well with the sample port of the colorimeter, to protect it from the interference of outside light. The colour of each sample was measured using a Colorflex HunterLab Spectrophotometer (Hunter Associates Laboratory Inc., Reston, VA, USA). Colour was evaluated using a diffuse illumination (D65 2° observer) with 8 mm viewing aperture and a 25 mm port size with the specular component excluded.

### 2.9. Instrumental Texture Measurement

Warner-Bratzler shear force values were determined from *M. longissimus dorsi* on the 1, 3, and 7 days of stored meat under refrigerated conditions by using Instron Texture Analyzer model 3343 device (USA) equipped with a Warner-Bratzler shear force system [18]. Shear force was perpendicular to the length of the 2 cm thick chops stakes which were parallel to the muscle fibre orientations, and force required to shear was recorded in kilograms. For each sample, eight to ten replicates were made, and a mean value was calculated for using in statistical analysis.

### 2.10. Sensory Evaluation

Eight semitrained panelists, staff of Istanbul University, Food Hygiene and Technology Department, who had previously participated in training sessions to become familiar with the sensory characteristics of meat [19, 20] were requested to score the sensory attributes (red-colour, animal odour intensity, tenderness, and general appearance acceptability) on the basis of nine-point hedonic rating scales. The scales included 1 = extremely unacceptable, 2 = very much unacceptable, 3 = moderately unacceptable, 4 = slightly unacceptable, 5 = between acceptable and unacceptable, 6 = slightly acceptable, 7 = moderately acceptable, 8 = very much acceptable, and 9 = extremely acceptable [21]. The panelists were trained in two separate sessions approximately 2 hours for the evaluation of selected attributes. Training sessions were conducted to acquaint panelists with the products and attributes to be evaluated and were followed by an open-discussion session to familiarise panelists with the attributes and the scale to be used.

Samples chosen for sensory analyses were served to the panel members, who were seated in individual booths in a temperature-controlled and light-controlled room (fluorescent lighting of 2000 lx; Philips 40 W Cool White), receiving a set of 8 samples served in a complete randomised order. Each sample was labelled, at random, with a two-digit code number [22]. Sensory panel was carried out triplicate in two sessions.

### 2.11. Statistical Analyses

The general linear model procedure (PROC GLM) of SPSS 13.0 program was used in the statistical analyzes of electrically stimulated lamb and goat meat [23]. Least squares procedures were used to analyze data for pH, water activity, WHC, drip loss, surface colour (CIE L*, a*, b* values), shear force, and sensory characteristics. The model used in the analyses of these characteristics included the fixed effects of ES and aging period. Significance of differences was defined as *P* < 0.05, and paired Student *t*-tests were used for comparison of the means in both animals.

## 3. Results and Discussion

The pH values obtained from carcass halves through the measurements at the 1, 3, 6, and 24 h are given in Table 1. In all groups treated with various levels of voltage, a considerable pH decrease relating the control group was observed in lamb and goat carcasses, and difference among the results of the groups was found statistically significant (*P* < 0.05).

According to these results, it was found that the pH values obtained from electrically stimulated lamb carcasses were lower than the goat ones, but not significantly different during the whole aging period (Table 6).

Similarly, it is reported in many studies that electrical stimulation accelerates the ATP and glycogen break down and causes a rapid pH decrease [3, 4, 9]. Kahraman and Ergun [4] stated that the pH values in *longissimus dorsi* muscle of lamb carcasses showed a significant correlation (*P* < 0.05) between ES and NES groups, but no significant differences were recorded between LVES (50 V, 100 Hz for 120 s) and MVES (100 V, 100 Hz for 120 s) treated lambs (*P* > 0.05). Ferguson et al. [8] determined that ES (300 V, 20 min) caused a significant difference in pH decline of electrically stimulated Merinos breed sheep. Similar results in pH have been reported by Polidori et al. [5] and Morton et al. [24] in lamb carcasses. Cetin and Topcu [9] reported also that the pH values of electrically stimulated goat carcasses were lower than the ones from the nonstimulated ones. Additionally, Biswas et al. [3] emphasized that significant improvement was observed on goat carcasses which were electrically stimulated with different voltages (35, 110, 330, 550, and 1100) and 50 Hz frequency. These results suggested that the ES treatment caused an acceleration of glycolysis and subsequent early rigor mortis development.

Water activity was enhanced with the range of voltage used and was higher than the control groups of both animal species (Table 2). The *a*_*w*_ value was decreased during the aging period depending on the ES used and was significantly different (*P* < 0.05) in lamb and goat meat (Table 6). Cetin and Topcu [9] also remarked that water activity values of electrically stimulated goat meat were higher than the control ones; however, any significant difference (*P* > 0.05) was observed between the control and the ES-treated lamb carcasses in a study conducted by Kahraman [25]. 

Water-holding capacity increased with the range of voltage used for ES (Table 3). The WHC values decreased with the aging period and differed in lamb and goat meat (Table 6). The effect of ES on water-holding capacity and protein denaturation is dependent on the muscle considered [16]. The number of components that bind water change postmortem with the loss of ATP drops in pH, proteolysis, and protein denaturation [15]. Kahraman and Ergun [4] determined that WHC was significantly greater for stimulated lamb carcasses (*P* < 0.05) and revealed that MVES (100 V, 100 Hz for 120 s) was more effective than LVES (50 V, 100 Hz for 120 s) only at 1 day of postslaughter period (*P* < 0.05). The authors indicated also that these results supported the theory that ES significantly increased drip loss. Cetin and Topcu [9] stated that WHC values of ES-treated goat decreased in aging period but were not significantly different to control ones. Similarly, Strydom et al. [26] determined that ES-treated (400 V) Cholaris breed lamb showed lower WHC than control group although no significant difference was observed. Contrary to these, Biswas et al. [3] reported that electrically stimulated Bengal goats showed significant differences (*P* < 0.05) with respect to water-holding capacity.

It has been observed that drip loss was enhanced with the range of voltage used and was higher than the control groups (Table 4). DL increased during the aging period depending on ES used and was significantly different (*P* < 0.05) in lamb and goat meat (Table 6). Cetin and Topcu [9] indicated that drip loss amount obtained from the ES-treated goat carcasses was greater and significantly different (*P* < 0.01) than control group. However, Bond et al. [27] stated that ES did not change the drip loss of 200 V applied sheep. Drip loss is formed over time as the meat is tenderised. When proteins degrade at postmortem, they release the binding water in muscles. ES ensures early rigor, protects the enzymes that tenderise meat, and promotes the protein degradation by resulting in increase in the drip loss [15, 16].

An evident improvement of shear force compared with control groups was observed in ES-treated lamb and goat meat, and difference between the results relating the animal groups was found important (*P* <0.001) during aging period (Table 5). Shear force of meat varied between lamb and goat, and lamb meat was defined more tender than goat one (Table 6). The improved tenderness associated with ES has been attributed to the prevention of cold shortening, increased proteolysis, and physical disruption of muscle fibers [10]. 

In similar studies performed on textural properties of small ruminant carcasses, the improving effect of electrical stimulation on tenderness was clearly determined [4, 5, 9, 11, 24, 28, 29]. Kahraman and Ergun [4] stated that ES applied carcasses were more tender than NES at 1 and 7 days of postslaughter period (*P* < 0.001) and added that significant differences were found between LVES and MVES (*P* < 0.05) applied lamb. These findings were in agreement with Morton et al. [24] in lamb carcasses. Yanar and Yetim [11] reported that 350 volt of electrical stimulation on 14 half-carcasses of 3–5 years old sheep improved the texture of *longissimus dorsi* muscle (*P* < 0.01) and made no considerable effect on the *semimembranosus* muscle. Similarly, Solomon and Lynch [30] emphasized that stimulated *longissimus dorsi* muscle of lamb carcasses had a significantly lower shear force value than nonstimulated one. Cetin and Topcu [9] indicated that shear force values obtained from the electrically stimulated goat carcasses were lower than nonstimulated ones. Polidori et al. [5] reported that ES remarkably affected shear force values of lamb carcasses and improved tenderness. Geesink et al. [29] notified that 1130 V of electrical stimulation positively affected shear force values of lamb meat. King et al. [10] observed that low-voltage ES was not effective on tenderness improvement in Cabrito carcasses but underlined that high-voltage ES was effective on tenderness at 1–3 days of postmortem. Additionally, Devine et al. [28] stated that electrically stimulated lambs were always more tender than nonstimulated lambs. 

**Figure 1:**
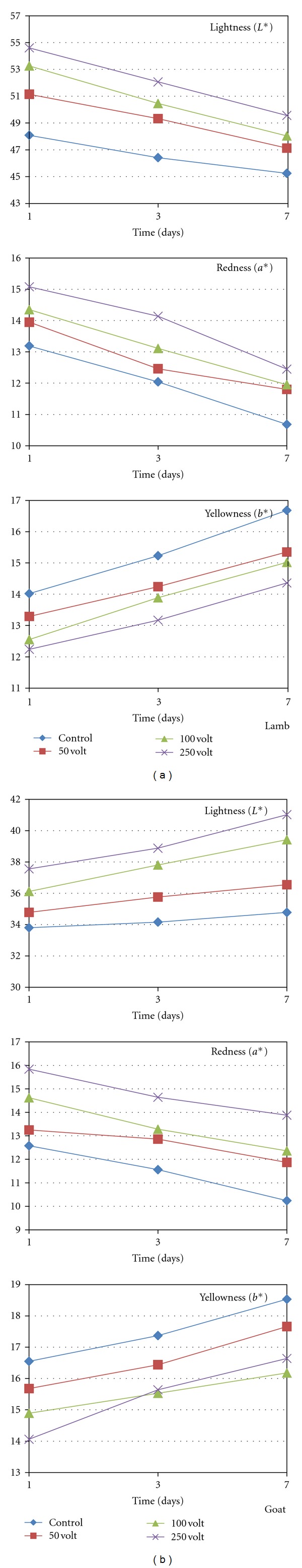
Instrumental colour (CIE L*, a*, b*) of lamb and goat meat aging at 4°C.

Various voltage treatments caused difference in instrumental colour values of lamb and goat meat compared with the control groups (Figure 1). The difference between the results of the animal groups has been significant during aging period at 4°C for L*, a*, and b* values, but not significant for b* values when compared with one another (Table 6). Lightness (L*) and redness (a*) increased with ES treatment, while a remarkable decrease was observed in yellowness (b*) of lamb and goat meat compared with controls. Similarly, in an observation of Kerth et al. [6] based on 5 different muscles, ES-treated lamb muscles were brighter with a better red colour than nontreated ones. In another study, 550 V of high-voltage electrical stimulation was applied to Cabrito carcasses, and ES increased a* and b* values of carcasses [10]. Kahraman and Ergun [4] explained that the initial colour parameters were not affected by ES (*P* > 0.05), but at the 7th day of aging period, significant differences were found in the redness (a*) values among the groups (*P* < 0.01). Cetin and Topcu [9] stated also that colour improvement was observed with ES treatment in goat carcasses, while significant differences were obtained (*P* < 0.01) only in L* value at the 7th day of aging. Opposite to the findings, Gadiyaram et al. [31] determined that ES had no significant effect on colour of castrated goat meat. This may be explained that ES reduces the colour stability, as defined by the rate of metmyoglobin accumulation in the surface layer of meat [32]. 

**Figure 2:**
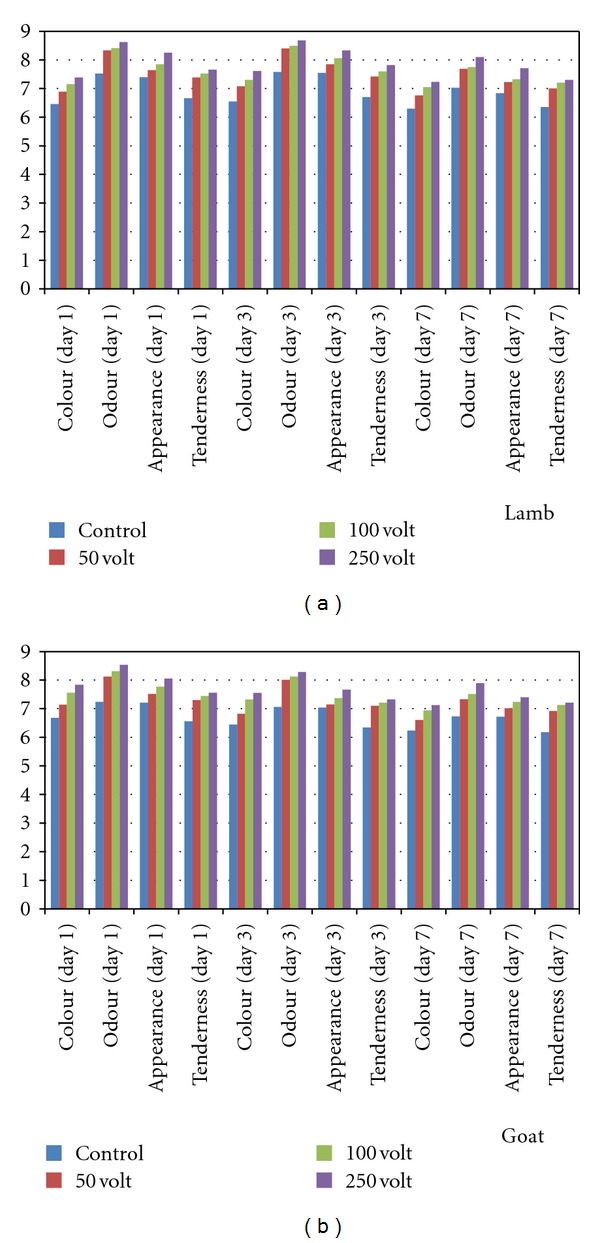
Sensory evaluation of lamb and goat meat during aging at 4°C.

Sensorial characteristics were improved with various levels of ES treatments. Significant difference was observed between ES and control groups (*P* < 0.05) during aging at 4°C, and red colour, odour intensity, and tenderness of both meats were particularly enhanced depending on voltage range used (Figure 2). Cetin and Topcu [9] found that sensorial characteristics were improved in the ES groups and were significantly different (*P* < 0.01) in raw samples. Yanar and Yetim [11] investigated that ES remarkably improved (*P* < 0.01) the tenderness of *longissimus dorsi* muscle in sheep meat. Contrary to these, Kerth et al. [6] evaluated that ES had no effect on sensorial characteristics of five different muscles of Hampshire × Rambouillet crossbred lambs (*P* > 0.05) but pointed out that the percentage of loin chops rated slightly tender or better was improved 30 to 34% by electrical stimulation (*P* < 0.05). 

## 4. Conclusion

Meat quality can be influenced by preslaughter and postslaughter factors. Electrical stimulation is one of the postslaughter methods to be used for increasing the meat quality. An obvious pH decrease and improvement in tenderness, colour, and sensorial characteristics can occur in lamb and goat meat by applying various voltage of electrical stimulation.

It is concluded that the electrical stimulation is a useful tool in the solution of cold-shortening problem of meat and in obtaining more tender and high-quality meat.

## Figures and Tables

**Table 1:** Mean and standard errors of pH values in lamb and goat meat during aging at 4°C.

Group	*n*	Lamb				Goat			
		1 h	3 h	6 h	24 h	1 h	3 h	6 h	24 h
Control (NES)	18	6.54 ± 0.032^a^	6.47 ± 0.013^a^	6.44 ± 0.052^a^	6.19 ± 0.022^a^	6.68 ± 0.49^a^	6.59 ± 0.55^a^	6.58 ± 0.51^a^	6.32 ± 0.40^a^
50 volt (LVES)	6	6.46 ± 0.023^b^	6.36 ± 0.042^b^	6.25 ± 0.033^b^	6.08 ± 0.032^b^	6.58 ± 0.21^b^	6.48 ± 0.17^b^	6.39 ± 0.39^b^	5.79 ± 0.61^b^
100 volt (MVES)	6	6.38 ± 0.023^b^	6.20 ± 0.042^c^	6.08 ± 0.033^c^	5.98 ± 0.032^b^	6.54 ± 0.21^b^	6.41 ± 0.17^b^	6.29 ± 0.39^b^	5.72 ± 0.61^b^
250 volt (HVES)	6	6.22 ± 0.023^c^	5.94 ± 0.042^d^	5.79 ± 0.033^d^	5.60 ± 0.032^b^	6.49 ± 0.21^c^	6.23 ± 0.17^c^	5.98 ± 0.39^c^	5.68 ± 0.61^b^
*P*	36	*	***	***	***	**	**	***	***

^a.b^ Values in column with different superscripts differ significantly (*P* < 0.05), (*): *P* < 0.05, (**): *P* < 0.01, (***): *P* < 0.001, (NS): not significant (*P* > 0.05).

**Table 2:** Mean and standard errors of water activity (*a*_*w*_) values in lamb and goat meat during aging at 4°C.

Group	*n*	Lamb			Goat		
		Day 1	Day 3	Day 7	Day 1	Day 3	Day 7
Control (NES)	18	9.60 ± 0.052^c^	9.58 ± 0.044^b^	9.55 ± 0.014^b^	9.63 ± 0.027^b^	9.60 ± 0.024^b^	9.58 ± 0.044
50 volt (LVES)	6	9.72 ± 0.035^b^	9.69 ± 0.061^ab^	9.64 ± 0.022^a^	9.75 ± 0.055^ab^	9.71 ± 0.035^ab^	9.69 ± 0.056
100 volt (MVES)	6	9.75 ± 0.035^b^	9.73 ± 0.061^a^	9.67 ± 0.022^a^	9.79 ± 0.055^a^	9.75 ± 0.035^a^	9.71 ± 0.056
250 volt (HVES)	6	9.79 ± 0.035^a^	9.71 ± 0.061^a^	9.64 ± 0.022^a^	9.81 ± 0.055^a^	9.77 ± 0.035^a^	9.72 ± 0.056
*P*	36	**	*	*	*	*	NS

^a.b^Values in column with different superscripts differ significantly (*P* < 0.05), (*): *P* < 0.05, (**): *P* < 0.01, (***): *P* < 0.001, (NS): not significant (*P* > 0.05).

**Table 3:** Mean and standard errors of water-holding capacity (WHC) values in lamb and goat meat during aging at 4°C (%).

Group	*n*	Lamb			Goat		
		Day 1	Day 3	Day 7	Day 1	Day 3	Day 7
Control (NES)	18	3.50 ± 0.057^c^	3.36 ± 0.214	2.81 ± 0.055^d^	3.62 ± 0.137	3.41 ± 0.250	2.90 ± 0.112^a^
50 volt (LVES)	6	3.57 ± 0.031^b^	3.25 ± 0.315	2.94 ± 0.082^c^	3.77 ± 0.243	3.48 ± 0.321	3.10 ± 0.210^b^
100 volt (MVES)	6	3.74 ± 0.031^a^	3.34 ± 0.315	3.08 ± 0.082^b^	3.89 ± 0.243	3.58 ± 0.321	3.16 ± 0.210^b^
250 volt (HVES)	6	3.88 ± 0.031^d^	3.51 ± 0.315	3.21 ± 0.082^a^	3.98 ± 0.243	3.67 ± 0.321	3.32 ± 0.210^c^
*P*	36	*	NS	**	NS	NS	*

^a.b^Values in column with different superscripts differ significantly (*P* < 0.05), (*): *P* < 0.05, (**): *P* < 0.01, (***): *P* < 0.001, (NS): not significant (*P* > 0.05).

**Table 4:** Mean and standard errors of drip loss (DL) values in lamb and goat meat during aging at 4°C (%).

Group	*n*	Lamb			Goat		
		Day 1	Day 3	Day 7	Day 1	Day 3	Day 7
Control (NES)	18	4.02 ± 0.054^c^	4.53 ± 0.041^b^	5.94 ± 0.108^c^	3.81 ± 0.104^c^	4.25 ± 0.413^b^	5.62 ± 0.180^c^
50 volt (LVES)	6	5.55 ± 0.033^b^	6.22 ± 0.024^ab^	6.72 ± 0.081^bc^	5.02 ± 0.227^b^	5.81 ± 0.234^ab^	6.50 ± 0.381^bc^
100 volt (MVES)	6	5.78 ± 0.033^b^	6.44 ± 0.024^a^	7.98 ± 0.081^b^	5.33 ± 0.227^b^	6.14 ± 0.234^a^	7.84 ± 0.381^b^
250 volt (HVES)	6	5.97 ± 0.033^a^	6.69 ± 0.024^a^	8.45 ± 0.081^a^	5.76 ± 0.227^a^	6.23 ± 0.234^a^	8.31 ± 0.381^a^
*P*	36	***	**	***	***	**	***

^a.b^Values in column with different superscripts differ significantly (*P* < 0.05), (*): *P* < 0.05, (**): *P* < 0.01, (***): *P* < 0.001, (NS): not significant (*P* > 0.05).

**Table 5:** Mean and standard errors of shear force values in lamb and goat meat during aging at 4°C (kg/cm^2^).

Group	*n*	Lamb			Goat		
		Day 1	Day 3	Day 7	Day 1	Day 3	Day 7
Control (NES)	18	14.62 ± 0.094^a^	11.65 ± 0.112^a^	10.64 ± 0.085^a^	15.23 ± 0.29^a^	12.46 ± 0.25^a^	11.56 ± 0.31^a^
50 volt (LVES)	6	13.71 ± 0.121^b^	10.57 ± 0.071^b^	9.44 ± 0.046^b^	14.43 ± 0.21^b^	11.39 ± 0.17^b^	10.73 ± 0.56^b^
100 volt (MVES)	6	11.88 ± 0.121^c^	9.49 ± 0.071^c^	8.76 ± 0.046^c^	12.18 ± 0.21^c^	10.54 ± 0.17^b^	9.64 ± 0.56^c^
250 volt (HVES)	6	10.56 ± 0.121^d^	8.73 ± 0.071^d^	8.11 ± 0.046^c^	11.06 ± 0.21^c^	9.12 ± 0.17^c^	8.58 ± 0.56^d^
*P*	36	***	***	***	***	***	***

^a.b^Values in column with different superscripts differ significantly (*P* < 0.05), (*): *P* < 0.05, (**): *P* < 0.01, (***): *P* < 0.001, (NS): not significant (*P* > 0.05).

**Table 6:** Functional parameters variation in lamb and goat meat during aging at 4°C.

Parameters	Storage time	Meat type	Mean	Std. error	*P* value
pH	1 h	Lamb	6.400	0.068	0.061
		Goat	6.573	0.040	
	3 h	Lamb	6.243^b^	0.115	0.006
		Goat	6.428^a^	0.076	
	6 h	Lamb	6.140^b^	0.138	0.005
		Goat	6.310^a^	0.125	
	24 h	Lamb	5.963	0.128	0.307
		Goat	5.878	0.149	

Shear force	Day 1	Lamb	12.693^b^	0.911	0.003
		Goat	13.225^a^	0.968	
	Day 3	Lamb	10.110^b^	0.637	0.016
		Goat	10.878^a^	0.705	
	Day 7	Lamb	9.238^b^	0.541	
		Goat	10.128^a^	0.649	0.023

WHC	Day 1	Lamb	3.673^b^	0.086	0.031
		Goat	3.815^a^	0.078	
	Day 3	Lamb	3.365	0.054	0.311
		Goat	3.535	0.057	
	Day 7	Lamb	3.010^b^	0.087	0.021
		Goat	3.120^a^	0.087	

*a*_*w*_	Day 1	Lamb	9.715^b^	0.041	0.005
		Goat	9.745^a^	0.040	
	Day 3	Lamb	9.678^b^	0.034	0.032
		Goat	9.708^a^	0.038	
	Day 7	Lamb	9.625^b^	0.026	0.046
		Goat	9.675^a^	0.032	

Drip loss	Day 1	Lamb	5.330^a^	0.445	0.016
		Goat	4.980^b^	0.418	
	Day 3	Lamb	5.970^a^	0.490	0.002
		Goat	5.608^b^	0.461	
	Day 7	Lamb	7.273^a^	0.575	0.000
		Goat	7.068^b^	0.616	

Lightness (L*)	Day 1	Lamb	51.778^a^	1.420	0.033
		Goat	35.565^b^	0.817	
	Day 3	Lamb	49.570^a^	1.191	0.026
		Goat	36.653^b^	1.053	
	Day 7	Lamb	47.498^a^	0.903	0.026
		Goat	37.943^b^	1.402	

Redness (a*)	Day 1	Lamb	14.143^a^	0.394	0.025
		Goat	14.073^b^	0.726	
	Day 3	Lamb	12.938^b^	0.457	0.029
		Goat	13.085^a^	0.635	
	Day 7	Lamb	11.720^b^	0.373	0.025
		Goat	12.088^a^	0.750	

Yellowness (b*)	Day 1	Lamb	13.025^b^	0.398	0.014
		Goat	15.295^a^	0.533	
	Day 3	Lamb	14.133	0.428	0.082
		Goat	16.245	0.426	
	Day 7	Lamb	15.353	0.488	0.137
		Goat	17.253	0.526	

^a.b^Values in column with different superscripts differ significantly (*P* < 0.05).
